# Asymmetry of the Ferroelectric Phase Transition in BaTiO_3_

**DOI:** 10.1002/adma.202516507

**Published:** 2025-12-10

**Authors:** Asaf Hershkovitz, Elangovan Hemaprabha, Rajesh Mandal, Pravin Kavle, Jamil Tanus, Maya Barzilay, Ching‐Che Lin, David Spirito, Semën Gorfman, Bo Wang, Ignacio J. Villar‐García, Neus Domingo, Long‐Qing Chen, Lane W. Martin, Yachin Ivry

**Affiliations:** ^1^ Technion Israel Institute of Technology Faculty of Materials Science and Engineering Technion City Haifa 3200003 Israel; ^2^ Technion Israel Institute of Technology Solid State Institute, Technion City Haifa 3200003 Israel; ^3^ Department of Metallurgical and Materials Engineering Indian Institute of Technology Madras Chennai 600036 India; ^4^ Department of Materials Science and Engineering University of California Berkeley CA 94720 USA; ^5^ Materials Sciences Division Lawrence Berkeley National Laboratory Berkeley CA 94720 USA; ^6^ Department of Materials Science and Engineering Wolfson Building for Mechanical Engineering Tel Aviv University Tel Aviv 6997801 Israel; ^7^ Materials Science Division Lawrence Livermore National Laboratory Livermore CA 94550 USA; ^8^ Department of Materials Science and Engineering The Pennsylvania State University University Park PA 16802 USA; ^9^ ICN2‐Institut Catala de Nanociencia i Nanotecnologia Campus UAB Bellaterra Barcelona 08193 Spain; ^10^ CELLS‐ALBA Synchrotron Radiation Facility Cerdanyola del Valles 08290 Barcelona Spain; ^11^ Center for Nanophase Materials Sciences Oak Ridge National Laboratory Oak Ridge TN 37830 USA; ^12^ Departments of Materials Science and Nano Engineering Chemistry and Physics and Astronomy and the Rice Advanced Materials Institute Rice University Houston TX 77005 USA; ^13^ Technion Israel Institute of Technology The Nancy & Stephen Grand Technion Energy Program (GTEP), Technion City Haifa 3200003 Israel

**Keywords:** origin of ferroelectricity, phase‐change materials, phase‐transition asymmetry, thermo‐elastic energy storage

## Abstract

Symmetry changes during phase transformations fundamentally determine the behavior of thermodynamic systems, governing phenomena as diverse as water evaporation, fermion condensation, and epidemic spreading. Phase transitions are conventionally divided into two classes, and this classification is typically assumed to remain invariant upon reversing the transition. Here, an asymmetric phase transformation is uncovered in the ferroelectric–paraelectric transition of single‐crystal BaTiO_3_, a model system for first‐order transitions. Under slow temperature variation (≤0.1 °C min^−1)^, thermodynamic, dielectric, and domain‐structure measurements reveal that the ferroelectric‐to‐paraelectric transition exhibits latent heat, phase coexistence, and a discontinuous order parameter, while these signatures are absent upon cooling. Complementary phase‐field simulations demonstrate similar behavior, attributing it to distinct elastic strain energy accumulation and release during heating and cooling. These findings reveal a first‐order character upon heating but a second‐order‐like behavior upon cooling, challenging the conventional paradigm of symmetric phase‐transition classification and suggesting new possibilities for ferroelectric‐based energy storage.

## Graphical abstract

Phase transitions are typically assumed to behave identically in forward and reverse. This work shows that in the ferroelectric material barium titanate this is not true: heating drives an abrupt, first‐order jump, while cooling gives a smooth, continuous change. This surprising asymmetry challenges standard classifications of phase transitions and opens new strategies to design novel energy‐storage devices.

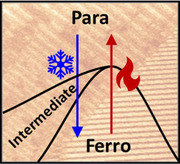

## 1 Introduction

Phase transformations define the manifestation of physical systems and provide us with vital information regarding both the system and its emergence. Such transitions are, in turn, defined by the universal class in which they fall. Following the generalized Ehrenfest classification,^[^ ^1^ ^]^ a first‐order transition is accompanied by latent heat, phase coexistence, and discontinuity of the first derivative of the free energy with respect to the thermodynamic variables, with a seminal example being water boiling at ambient pressure. On the other hand, there is no latent heat in a second‐order transition, and the first derivative of the free energy, which is often described by the order parameter of the system, which is continuous, as in the case of the transition that occurs at the critical temperature in type‐II superconductivity.^[^ ^2^ ^]^ It is commonly agreed that if the tuning parameter is varied slowly in a quasi‐static way, the phase transition is symmetric around the critical‐state variable (temperature, pressure, etc.). For instance, the liquid‐to‐vapor and vapor‐to‐liquid transitions in water are both first order. Nevertheless, a rigorous examination of the phase‐transition symmetry is still lacking.

Barium titanate (BaTiO_3_) is a prototypical system in which there is a phase transition that is accompanied by spontaneous symmetry breaking,^[^ ^3^ ^]^ as well as a first‐order displacive transition,^[^ ^4^ ^]^ thus lending itself as an excellent platform to examine the symmetry of phase transitions. Early observations showed that, similar to other first‐order systems, the phase transition in BaTiO_3_ is accompanied by a thermal hysteresis such that the Curie temperature varies between cooling and heating,^[^ ^5^, ^6^ ^]^ while the transition remains first order on both sides. The existence of thermal hysteresis effectively broadens or “slows down” the transition, hence providing easier access to examine the transition dynamics in detail. Moreover, it was proposed that the thermal hysteresis is associated with elastic‐energy storage,^[^ ^6^ ^]^ thus utilizing BaTiO_3_ for energy‐storage devices that are similar in concept to supercooling^[^ ^7^ ^]^ and are technologically attractive.

Following the experimental observations by Helen Megaw^[^ ^8^ ^]^ and the theoretical model proposed by Cochran,^[^ ^9^ ^]^ ferroelectric transitions were initially understood to arise purely from displacive mechanisms. These transitions are characterized by diffusionless, collective atomic displacements. However, more recent studies based on X‐ray scattering, first‐principles calculations, and computational models suggest that ferroelectric transitions involve a combination of displacive and diffusive components. Therefore, considering the ferroelectric‐transitions as a picture of a simple displacive transition is somewhat naive because the cubic‐tetragonal lattice mismatch is energetically expensive.^[^ ^10^ ^]^ This is reminiscent of the case of steel that undergoes a martensitic transition upon cooling and can accommodate the cubic‐tetragonal mismatch by rapid thermal‐energy transfer.^[^ ^11^ ^]^ In steel, however, the resultant phase is only preserved by rapid freezing,^[^ ^12^ ^]^ whereas for BaTiO_3_, the ferroelectric tetragonal structure is considered stable thermodynamically.^[^ ^13^ ^]^ Special attention has been given to BaTiO_3_ thin films, in which strain is tuned with substrate selection, giving rise to a vivid ongoing discussion of the exact phase diagram in the thermal‐elastic energy space.^[^ ^10^ ^]^ Based on such diagrams, the transition between cubic and tetragonal structures in strained films is mediated by a foreign phase in many cases.^[^ ^10^, ^14^ ^]^ This foreign phase may be a monoclinic crystallographic phase,^[^ ^14^ ^]^ but is sometimes considered as a certain mixture of local elastic domains and not necessarily a different lattice structure globally.^[^ ^15^ ^]^ The existence of such an intermediate phase is often considered to change the nature of the transition from first order to second order in thin films.^[^ ^14^ ^]^ Yet, although BaTiO_3_ has been researched thoroughly in the past century, the exact mechanism of the phase transition, even in bulk crystals, is still debated. Moreover, the origin of the thermal hysteresis that puzzled earlier researchers has yet to be addressed, and so are the effects of electric and elastic energy on this hysteretic behavior.^[^ ^16^ ^]^

Here, direct observations were used to demonstrate that the phase‐transition process in BaTiO_3_ is asymmetric at the quasi‐static limit (slow temperature variation) with a first‐order‐like transition upon heating and a second‐order‐like transition upon cooling. The origin of this asymmetry is also demonstrated by means of direct observations that show that a mediating phase exists only during the paraelectric (cubic)‐to‐ferroelectric (tetragonal) transition and not with the reverse transition. The structure and properties of single‐crystal BaTiO_3_ were characterized with high accuracy of temperature to reveal the exact phase‐transition mechanism. Using differential scanning calorimetry (DSC) with low cooling and heating rates (0.1 °C min^−1^), it was demonstrated that the cooling and heating transitions are asymmetrical. Latent heat was observed clearly upon heating from the tetragonal phase to the cubic phase, but the opposite transition upon cooling did not show a similar behavior. Similar asymmetry was observed in both impedance and polarization‐electric field (*P*‐*E*) hysteresis loop measurements, which revealed a sharp transition upon heating and a more gradual transition when the crystal was cooled down from the paraelectric to the ferroelectric phase. Increasing the temperature variation rate by an order of magnitude (1 °C min^−1^) resulted in a symmetric transition in both heat flow and electric characteristics, similar to results in the literature.^[^ ^17^, ^18^ ^]^ The surface charge, which compensates for the intrinsic ferroelectric polarization, was also found to change asymmetrically during the transition. Piezoresponse force microscopy (PFM) with high‐precision temperature control (0.1 °C min^−1^) was performed to illustrate that the tetragonal‐to‐cubic transition occurs abruptly with co‐existence between the two phases, while the cubic‐to‐tetragonal transition is accompanied by a foreign domain structure that mediates between the paraelectric cubic state and the final domain structure. Lastly, the asymmetric transition and the existence of an intermediate state at the cubic‐to‐tetragonal transition were confirmed by acquiring high‐resolution X‐ray scattering reciprocal space maps every 0.05 °C around the transition. Phase‐field simulations on multi‐domain BaTiO_3_ also support the observed thermal asymmetry of the order parameter.

## 2 Results and Discussion

### 2.1 Asymmetric Heat Flow and Dielectric Permittivity During the Ferroelectric‐Paraelectric Transition in BaTiO_3_

**Figure 1:**
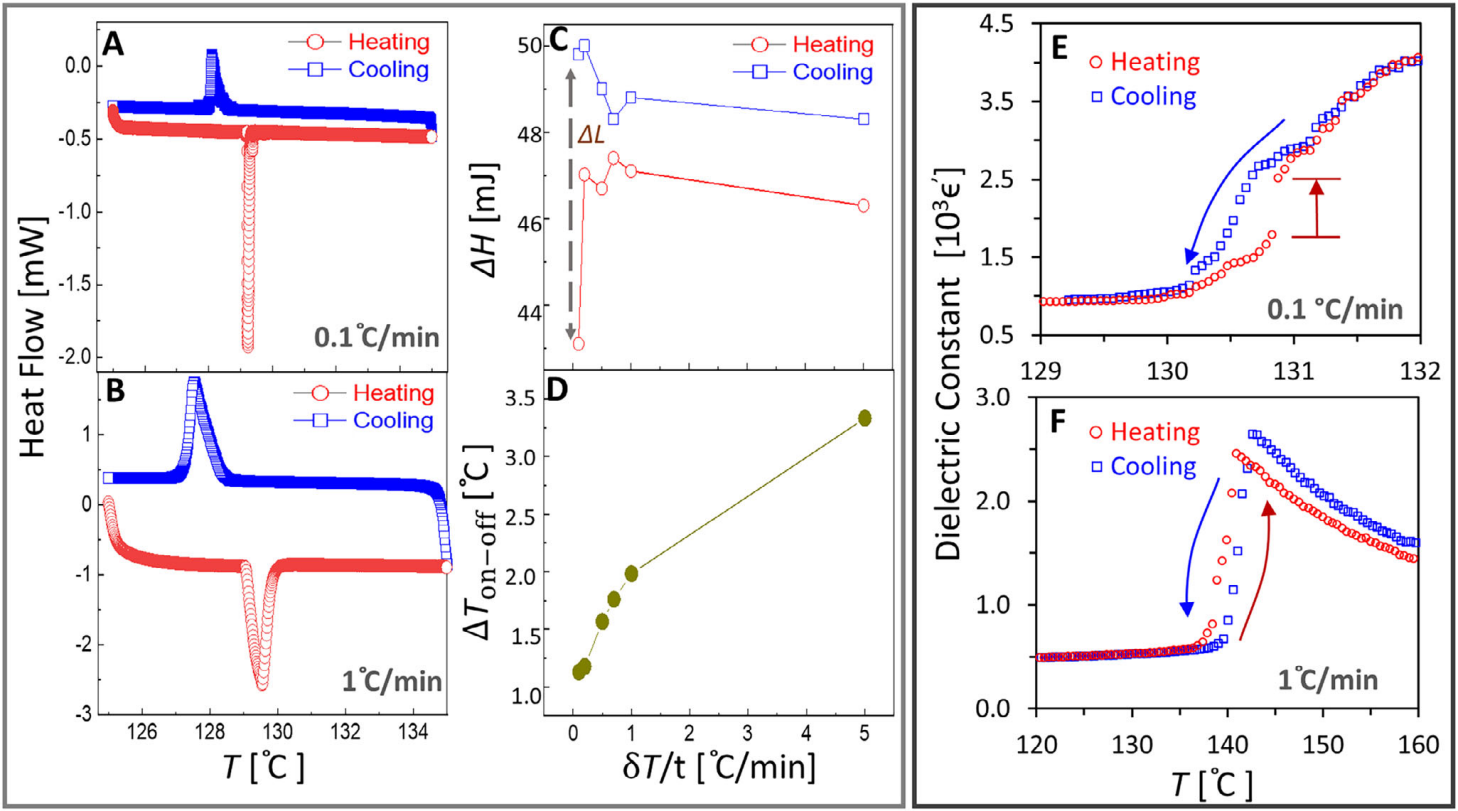
Asymmetric latent heat and dielectric permittivity under a slow temperature variation. Differential scanning calorimetry (DSC) heat flow during A) slow, 0.1 °C min^−1^, B) fast, 1 °C min^−1^ (heating and cooling. C) Enthalpy change (Δ*H*) showing a large difference in latent heat for slow temperature variation (Δ*L* = 6.6 mJ) and D) difference in the transition temperatures (Δ*T*_on − off_) during heating and cooling for different temperature variation rates (δ*T*/*t*, *t* is time). E) Dielectric permittivity values measured during slow (0.1 °C min^−1^) and F) fast (1 °C min^−1^) heating and cooling, showing a smooth transition during slow cooling, and some abrupt kinks during slow heating, while also revealing a large difference (about twice) at the transition temperature for fast temperature variation. Additional dielectric measurements at multiple scan rates (0.05, 0.1, and 0.5 °C min^−1^), showing rate‐dependent threshold for the evolution of phase transition characteristics, are provided in Figure SI2 (Supporting Information).

The temperature dependence of heat flow and enthalpy change during the transition was first evaluated with DSC. **Figure** **1**A shows that when the temperature variation is accomplished slowly (0.1 °C min^−1^), a sharp, high, and narrow peak in heat flow is observed at the ferroelectric (tetragonal)‐to‐paraelectric (cubic) transition (*T*_on_ = 129.2 °C upon heating). The reverse paraelectric‐to‐ferroelectric (cooling) transition occurred at a lower temperature (*T*_off_ = 128.1 °C), and the peak in heat flow is much lower, broader, and smoother. Figure 1B shows that repeating this experiment at a higher temperature‐variation rate (1 °C min^−1^) resulted in a more symmetric specific‐heat and enthalpy behavior in both directions. Extracting the difference in enthalpy during the transition also revealed a large difference between the heating and cooling processes (Figure 1C). This strong asymmetry, both in peak width and enthalpy, indicates the existence of latent heat that is characteristic of a first‐order transition upon heating and a second‐order‐like behavior upon cooling. Figure 1D summarizes the effects of temperature‐variation rate on the thermal hysteresis (Δ*T*_on − off_), and the corresponding DSC heat flow measurements are provided in Figure SI1 (Supporting Information).

The transition asymmetry was then characterized independently through impedance measurements. Figure 1E shows that for slow temperature variation (0.1 °C min^−1^), the dielectric permittivity evolved more smoothly upon cooling during the phase transition, while the heating process was accompanied by an abrupt change. Moreover, a ≈1 °C difference was observed between the two transition temperatures. Figure 1F shows that fast temperature variation (1 °C min^−1^) resulted in a sharp jump and not a gradual change in the dielectric permittivity during both cooling and heating, which is similar to the well‐documented behavior in the literature.^[^ ^17^, ^19^ ^]^

### 2.2 Asymmetric Polarization Dynamics and Intermediate Polarization State During the Ferroelectric‐ Paraelectric Phase Transition

**Figure 2:**
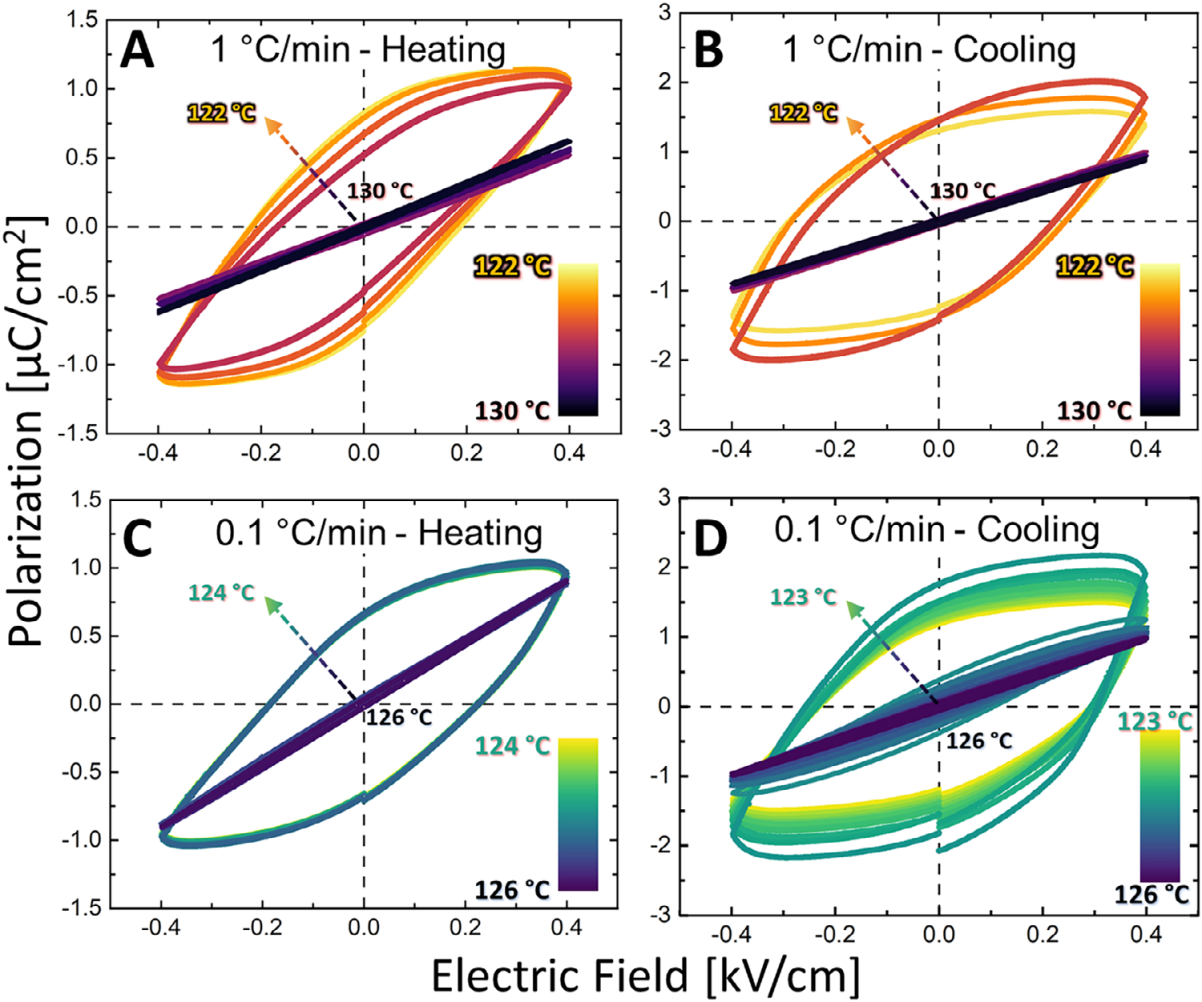
Asymmetric P‐E loop dependence during a slow temperature variation. Temperature dependence of the *P‐E* loop for fast (1 °C min^−1^) A) heating and B) cooling. Slow (0.1 °C min^−1^) C) heating and D) cooling.

Next, the temperature dependence of the order parameter was examined. Variable‐temperature *P*‐*E* hysteresis loop measurements were performed to evaluate the dynamics of the polarization (the primary order parameter) in capacitor‐based devices. **Figure** **2**A,B shows the abrupt disappearance and reappearance of the hysteresis loop at the ferroelectric‐to‐paraelectric (heating) and paraelectric‐to‐ferroelectric (cooling) transitions upon rapid temperature variation (1 °C min^−1^). Figure 2C shows that similar behavior was observed during slow heating (0.1 °C min^−1^). Nevertheless, upon slow cooling from the paraelectric state (Figure 2D), the typical linear paraelectric curve did not transform immediately to a well‐behaved hysteresis. Rather, a small, distorted hysteresis loop emerged (*T* = 124.5 ^○^C). The hysteresis curve of this intermediate state opened gradually, and only then, at a lower temperature (*T* = 124 ^○^C), transformed to the typical hysteretic behavior.

### 2.3 Asymmetric Local Domain Dynamics and Appearance of Intermediate Domain States

**Figure 3:**
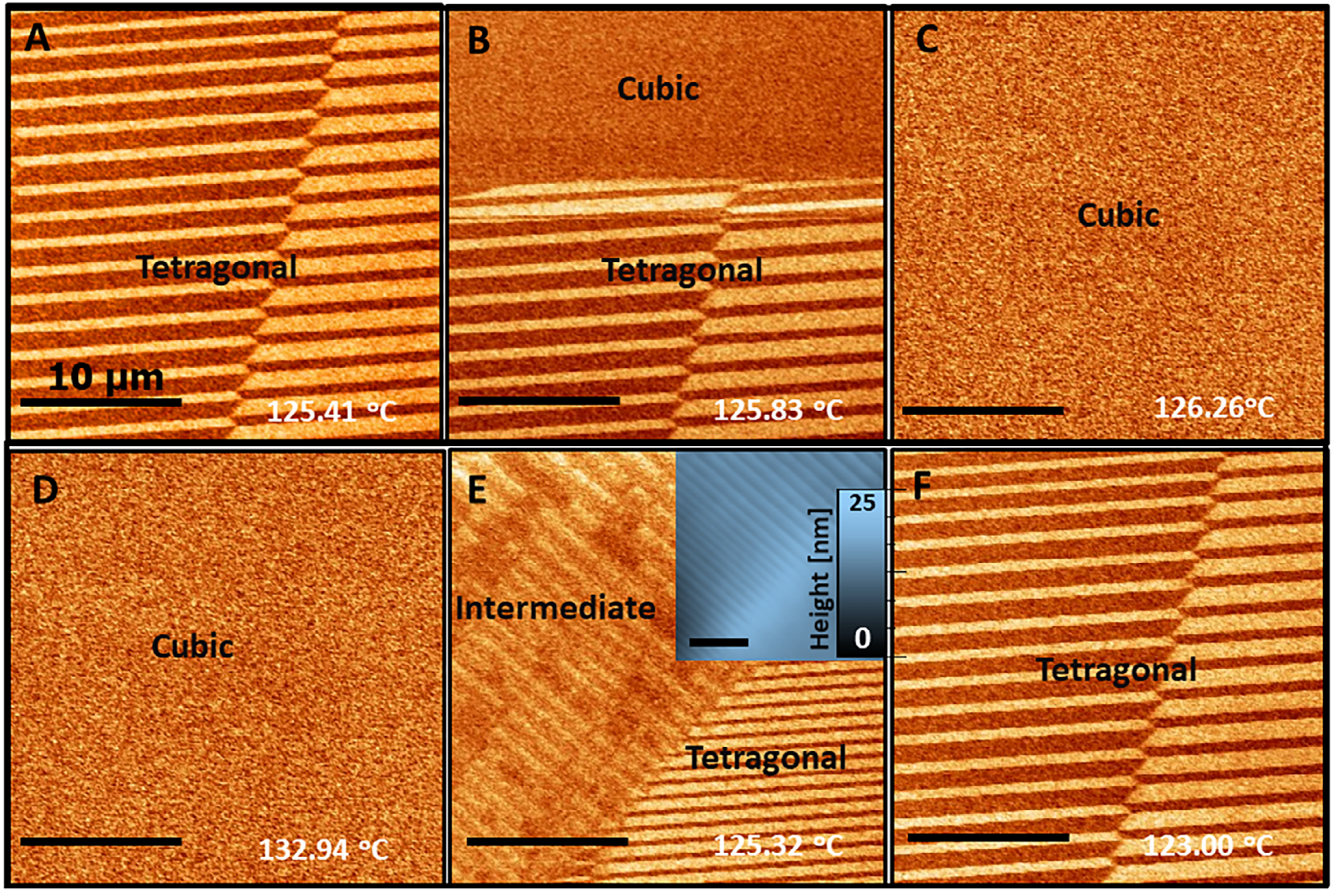
Domain dynamic asymmetry during slow temperature variation (0.1 °C min^−1^) through the ferroelectric‐paraelectric transition. PFM lateral amplitude signal upon heating shows that the ferroelectric‐paraelectric transition takes place from the A) tetragonal phase through B) a paraelectric‐ferroelectric phase‐coexistence at the transition to C) the final paraelectric cubic phase. Upon cooling from D) the paraelectric phase, no paraelectric‐ferroelectric phase coexistence was observed. Rather, E) a coexistence of two domain phases was observed, before F) one domain phase [the striped on the right, in (E)] became the tetragonal domains of the ferroelectric phase. Complementary PFM phase signals are given in Figures SI3 and SI4 (Supporting Information).

Enthalpy, dielectric permitivity, and polarization provide a clear picture of the transition asymmetry at the macroscopic scale. The macroscopic properties of ferroelectrics originate from the collective behavior of the microstructure and domain dynamics. Thus, additional insights are needed from the microstructure and mesoscopic domain scales. The domain dynamics during the transition were observed here by means of PFM with precise temperature control (0.1 °C min^−1^).^[^ ^20^ ^]^ **Figure** **3**A–C shows striped *a*_1_‐*a_2_* and *a*_2_‐*a_1_* ferroelastic domains at 125.4 °C. Upon heating, these domains vanish completely at 126 °C, but a coexistence of the striped domains with a region of the material that has already undergone the transition to the paraelectric state is observed between these two temperatures (at 125.83 °C, Figure 3B). Similar phase coexistence was not observed upon cooling (Figure 3D–F). Rather, while the end ferroelastic phase demonstrated similar domain distributions to the original structure (123 °C) in agreement with recent observations,^[^ ^21^ ^]^ an intermediate domain structure was observed at 125.32 °C. The striped domains of the intermediate phase align 45° with respect to the original domains and are also observed in the topography (inset, Figure 3E). That is, the PFM observations confirmed the asymmetry of the ferroelectric tetragonal‐to‐paraelectric cubic phase transition and revealed that while the ferroelectric‐to‐paraelectric transition comprises phase coexistence, as expected from a first‐order transition, the paraelectric‐to‐ferroelectric transition is accompanied by the introduction of a foreign domain phase that mediates between the paraelectric and the final tetragonal domain phases. To verify the reproducibility of these observations, similar results were observed in several repeating experiments.

### 2.4 Asymmetric Lattice‐Parameter Dynamics and the Appearance of an Intermediate State

**Figure 4:**
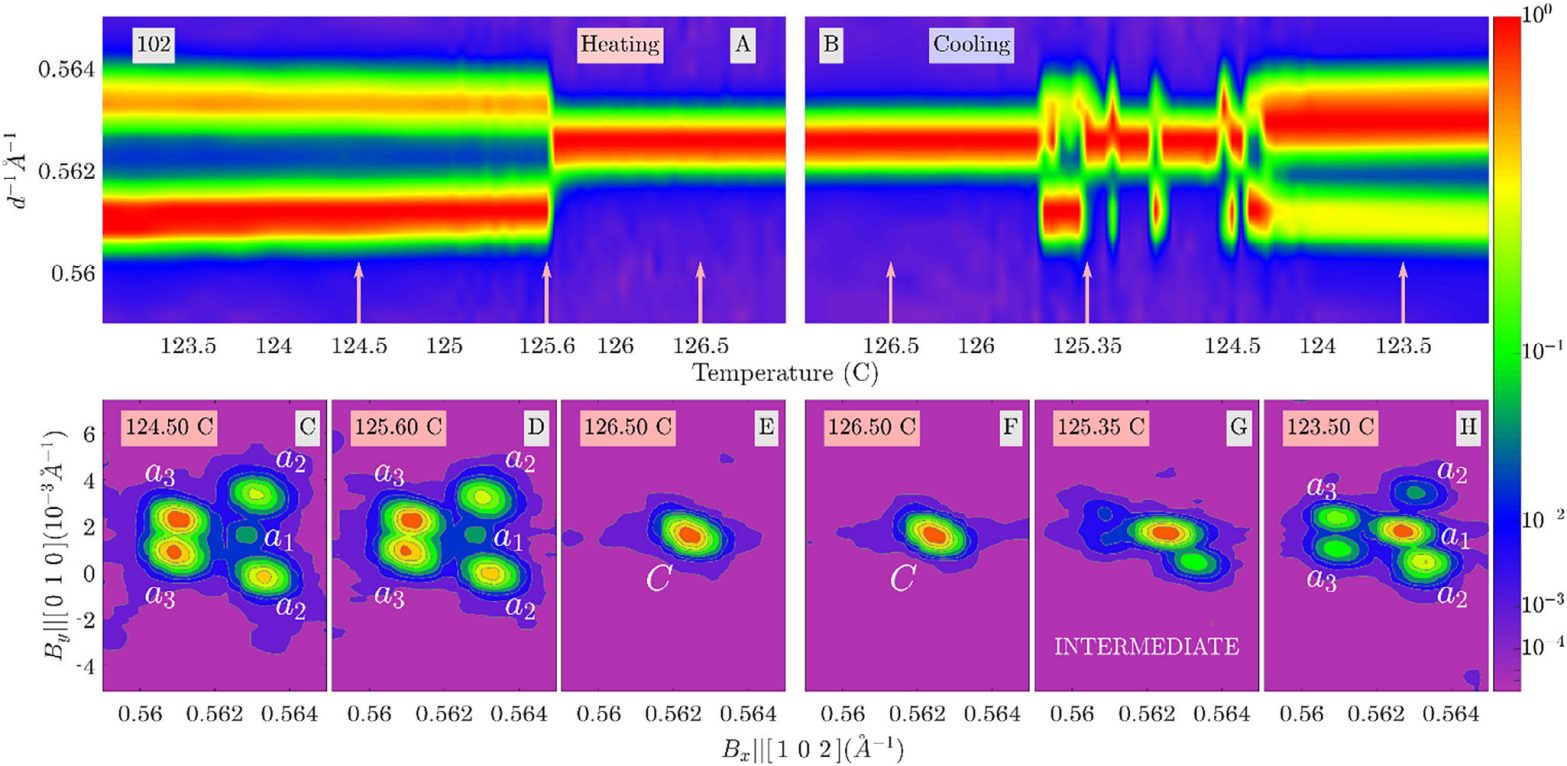
Reciprocal space maps around the ferroelectric transition in single‐crystal BaTiO_3_. A) Integrated inter‐planar distance intensity peaks as a function of temperature during heating and B) cooling the material around the ferroelectric‐paraelectric phase transition around the 102 Bragg reflection. C–E) Individual RSM snapshots upon heating and F–H) cooling at certain temperatures (designated in C–F and highlighted in A‐B) around the transition reveals an asymmetric behavior. The individual sub‐peaks are assigned to the tetragonal *a*_1_, *a*_2_, and *a*_3_ domains following the method described in ref. [23]. Panel (G) is explicitly marked as corresponding to the intermediate phase. A and B present the temperature dependence in the form of *I*(*d* ^−1^,*T*), which was obtained by the corresponding integration of the data. Here, $d^{-1}={\left(B_{x}^{2}+B_{y}^{2}+B_{z}^{2}\right)}^{\frac{1}{2}}$.

Next, after the phase‐transition asymmetry was backed up by observations of the domain evolution, the structure was examined by means of a series of 3D reciprocal space volumes (RSVs) which represent the diffraction intensity distribution *I*(B_x_,B_y_,B_z_) around certain Bragg peaks of BaTiO_3_. Here, B_x_,B_y_, and B_z_ are the Cartesian coordinates of the scattering vector: the coordinate system is chosen so that B_x_ ≈ |B| = *d* ^−1^ with *d* being an interplanar distance corresponding to the measured Bragg peak. Below the phase‐transition temperature, such Bragg peaks split into several components due to the presence of ferroelastic domains. These observations were made via variable‐temperature (VT) X‐ray diffraction (XRD) reciprocal space mapping (RSM) on a custom‐built four circle diffractometer (Experimental Section).^[^ ^22^ ^]^ The macroscopic phases around the ferroelectric‐paraelectric phase transition were inspected directly from a set of 2D projections of a 3D intensity distribution. **Figure** **4** shows VT‐XRD‐RSM with < 0.05 °C min^−1^ temperature variation rate. Figure 4A (heating) and B (cooling) are *I*(*d* ^−1^) curves, obtained by the integration of diffraction intensity distribution along B_y_ and B_z_; such curves are usually measured using conventional powder‐diffraction techniques. These results illustrate an abrupt tetragonal‐to‐cubic transition during heating, similar to the transition documented in the literature. At the same time, Figure 4B shows that the cubic‐to‐tetragonal transition during cooling includes a ≈1 °C temperature range where neither a single cubic nor a single tetragonal phase fits the data satisfactorily. In addition, the RSM studies allow for the examination of the corresponding phase at each temperature. Figure 4C,D,H show the assignment of the sub‐peaks within the RSM to the *a*_1_,*a*_2_,*a*_3_ tetragonal domains, according to the method described previously.^[^ ^23^ ^]^ Merging all the sub‐peaks into a single peak, Figure 4E,F indicates the transition to the cubic phase. Figure 4G displays an RSM at the “intermediate” temperature, showing that the sub‐peak topology is different from those at the “tetragonal” or “cubic” ranges. Previous studies at higher heating/cooling rates (≈10 °C min^−1^)^[^ ^21^, ^24^ ^]^ did not reveal such asymmetry, indicating that the intermediate phase and the associated transition characteristics emerge only under slow, near‐equilibrium conditions. The complete temperature‐dependent XRD‐RSM data are provided in Videos S1–S8.

### 2.5 Thermodynamic Analysis and Phase‐Field Simulations Show an Asymmetric Ferroelectric‐Paraelectric Phase Transition

**Figure 5:**
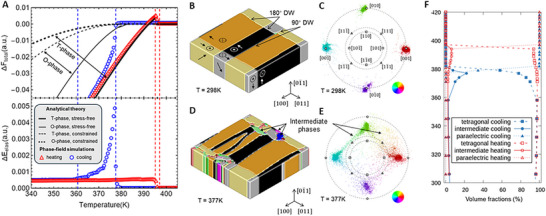
Thermodynamic analysis and phase‐field simulations of phase transitions in BaTiO_3_ under slow cooling and heating. A) The calculated total free energy density *ΔF*_total_ and elastic energy *ΔE*_elas_ (normalized to arbitrary units) as a function of temperature based on the analytical model of monodomain T‐phase or O‐phase BaTiO_3_ under stress‐free or constrained states and the phase‐field simulations of inhomogeneous multidomain BaTiO_3_ under macroscopic stress‐free conditions. B) The simulated domain structure of BaTiO_3_ at equilibrium at *T* = 298 K. The arrows denote the spontaneous polarization vector of the tetragonal domains. C) The pole figure showing the stereographic projection of local polarization vectors corresponding to the domain structure in (B). The low‐index directions are labeled in the pole figure, and the color scheme in the lower‐right corner is used to distinguish the orientation of the polarization vectors. D,E) Similar plots corresponding to (B,C) but for *T* = 377 K in the cooling simulation. The intermediate phases are denoted. F) The simulated volume fractions of tetragonal, intermediate, and paraelectric phases during heating and cooling.

Once the asymmetric behavior at the phase transition was established experimentally, both at the macroscopic and microscopic scales, a deeper discussion about the origin of this effect was needed. To understand the asymmetric behavior of the phase transitions and the possible mechanism for the formation of the intermediate phase upon cooling, we performed thermodynamic analysis based on the Landau–Ginzburg–Devonshire (LGD) theory^[^ ^25^, ^26^, ^27^, ^28^ ^]^ for homogeneous monodomain BaTiO_3_ and phase‐field simulations for inhomogeneous multidomain BaTiO_3_. For stress‐free monodomain BaTiO_3_, we found a much wider undercooling (≈11 K) than the superheating (≈3 K), which results in a more pronounced discontinuity of the dielectric permittivity during cooling than heating (Figure SI5, Supporting Information) and agrees well with experimental results of DSC and dielectric permittivity measurements (Figure 1). The bulk BaTiO_3_ crystal in experiments is likely to be inhomogeneous strained, e.g., stress‐free in the proximity of surfaces while constrained in the interiors. **Figure** **5**A shows that when the hydrostatic strain is considered, the first‐order transition of monodomain BaTiO_3_ becomes second‐order, and the orthorhombic phase becomes thermodynamically stable, suggesting that strains might account for the change of phase‐transition behavior and formation of intermediate phases. The phase‐field simulations of an inhomogeneous multidomain BaTiO_3_ bulk crystal also show asymmetry in terms of the free energy variations and the domain structure evolution during heating and cooling (Figure 5B–E). We tracked the volume fractions of cubic, tetragonal, and other possible phases during the transitions and identified up to 20% intermediate phases emerging only during the cooling process (Figure 5F). Real space mapping of the intermediate phases (Figure 5D) and corresponding pole figures of the local polarization vectors suggest the intermediate phases nucleate near the domain walls of tetragonal domain variants and are of monoclinic symmetry. Meanwhile, the emergence of the intermediate phase upon cooling is accompanied by an accumulation and relaxation of the elastic strain energy, which is absent upon heating (lower panel of Figure 5A). According to classical nucleation theory, the critical nucleus size decreases with increasing temperature deviation from the equilibrium transition point, leading to accelerated kinetics at larger driving forces. However, at the quasi‐equilibrium limit achieved in our slow temperature sweeps, the observed asymmetry between cooling and heating transitions may be rationalized within the classical nucleation‐and‐growth framework. The simulation results not only reproduce the asymmetric behavior of the phase transitions seen in the experiments but also provide evidence for the formation of intermediate phases as a mechanism to accommodate the nucleation of ferroelectric tetragonal domain variants within the paraelectric cubic matrix. Detailed discussion about the consistency with the different experimental results is given in Section SI5.

The stripes of the intermediate‐domain phase that emerge during cooling form a 45° angle with the striped ferroelastic domains of the final tetragonal phase (Figure 3E; Figure SI6, Supporting Information). Following the analysis of Marton et al. within the LGD framework,^[^ ^29^ ^]^ this angle may refer to 90° tetragonal domain walls with a normal along the $[\frac{1}{\sqrt{2}}-\frac{1}{\sqrt{2}}\;0$]. The crystallographic phase cannot be determined easily with PFM, but the height difference of the intermediate phase striped domains (inset, Figure 3E) suggests that these are *a*_1_/*c* domains, and the tetragonal bundle domains^[^ ^21^ ^]^ (Figure 3E) comprise *a*_1_/*a*_2_ domains, while the end domains (Figure 3F) are *a*_1_/*a*_2_‐*a*_2_/*a*_1_ bundle domains. Nevertheless, the XRD data (Figure 4G) indicate a possibly more complex structure, which might be an adaptive phase consisting of tetragonal polydomains or a crystallographic phase with lower symmetry (e.g., monoclinic). It has been shown^[^ ^30^, ^31^, ^32^, ^33^ ^]^ that the paraelectric‐to‐ferroelectric phase boundary of BaTiO_3_ exhibits a well‐defined orientation which deviates by 5° from the (110) plane and is approximately parallel to the (056) plane. Periodic‐twin domains consisting of two ferroelastic domain variants with a ratio ≈2:1 have been seen in the tetragonal phase,^[^ ^34^ ^]^ suggesting that the habit plane is dominated by the elastic‐strain energy to accommodate the mechanical compatibility at the interfaces. The PFM images (Figure 3A,B,F) also demonstrate twin domains with a ≈2:1 ratio. Particularly, the twin domains occur with two bundles, the junction of which corresponds to the type‐III super‐domain boundaries.^[^ ^34^ ^]^ To further confirm the strain effect, variable‐temperature domain imaging was done for a single‐crystal BaTiO_3_ lamella^[^ ^35^, ^36^ ^]^ (Section SI7). Here, domain rotation was observed for a short temperature range upon cooling the relaxed sample. On the other hand, when strain was induced via a thermal shock, the rotated domains remained stable for a very broad temperature range before reorienting to the final tetragonal domain phase (Figures SI7 and SI8, Supporting Information). These results agree with the broad temperature range in which the intermediate phase is stable in highly strained materials (Section SI8), consistent also with combined experimental‐simulation results. Notably, significant strain accumulation—and hence stabilization of the intermediate phase—is realized only under slow heating and cooling rates, where the system remains near thermodynamic equilibrium and strain states of 0.2–0.5% can be sustained. This behavior can be further clarified as follows. Although the overall free energy of a certain structure (say, cubic) is more stable for a given temperature (say, at *T*c), the material will not consume this new symmetry unless it is possible for that phase to evolve dynamically. Specifically, a structural transition from cubic to tetragonal requires that the two phases can match, so that the resultant phase will nucleate. However, the cubic‐tetragonal in BaTiO_3_ mismatch is rather large.^[^ ^10^ ^]^ Thus, introducing external energy, such as strain or heat, can help overcome that mismatch barrier. In the absence of such an additional energy source, an intermediate phase (e.g., monoclinic) can have a lower energy, which match with both the higher and lower symmetry phases and becomes energetically favorable. Note that because the domain structure reflects the short‐range ordering, it is a good representation of the overall macroscopic structure in the case when such an intermediate phase is introduced.

## 3 Conclusion

Finally, the asymmetric transition reported in this work may be considered as another similarity between the phase transition in BaTiO_3_ and the martensitic transition. That is, there has been substantial literature that discussed the asymmetry in heat flow between cooling and heating around the phase transition in shape memory alloys.^[^ ^37^ ^]^ While martensitic transitions occur typically during rapid temperature variation and help stabilize metastable phases, the current work shows that the transition in BaTiO_3_ is actually stabilized by slow temperature variation. Yet, similar to the current work, the asymmetry in shape memory alloys is attributed to mechanical energy that is stored in the system and is released in one direction only.^[^ ^37^, ^38^ ^]^ Further, the presence of second‐order‐like phase transitions during the cooling cycle suggests that the transformation proceeds without classical nucleation and growth mechanisms. Once initiated, such transitions may propagate continuously, effectively lowering the energy barrier for domain reorientation. This can lead to faster switching dynamics, which is particularly advantageous for non‐volatile ferroelectric memory applications. Thus, we encourage detailed high‐temperature‐resolution mesoscopic and microscopic re‐examination of the crystal structure and functional properties dynamics in these materials, also during slow temperature variation phase transitions.

It is reported that temperature‐driven and electric field‐driven domain formation are similar. Therefore, the systems of the intermediate phase for domain formation may be used for stabilizing the fast switching of the electric field domain. Further, the presence of an intermediate phase has been observed in a seminal material like BaTiO_3_, which triggers the exploration of such a behavior in other ferroelectric materials also. In BaTiO_3_, the threshold for observing a second‐order‐like phase transition was when the material was cooled at very low ramp rates, such as 0.1 °C min^−1^. However, in other materials, this window can be larger, and hence helps in leveraging the phenomenon for other materials also.

Intermediate phases have also been proposed in ferromagnetic systems, particularly in the context of tunnel magnetoresistance‐based magnetic random‐access memory (TMR‐MRAM), where such phases can facilitate reliable and rapid switching. These observations suggest that the presence and role of intermediate states in governing field‐driven switching dynamics may be a more general phenomenon across ferroic systems, and could be systematically investigated for functional optimization in memory technologies. Previous studies at higher heating/cooling rates (≈10 °C min^−1^)^[^ ^21^, ^24^ ^]^ did not reveal such asymmetry, indicating that the intermediate phase and the associated transition characteristics emerge only under slow, near‐equilibrium conditions.

## 4 Experimental Section

### Samples

More than four different commercial single crystal BaTiO_3_ (5 × 5 × 1 mm^3^) samples from MaTecK, SurfaceNet, and MTI Corporation were used for the measurements to ensure the universal nature of the phenomenon. For instance, the samples used in XRD‐RSM, PFM, DSC, and dielectric measurements are not identical samples, and they are distinct. Note that while the universal trend of heating/cooling asymmetry was observed in all experiments, as a matter of fact, in a multi‐institute study with independent experiments, the calibration for transition temperature in different methods varies.

### Temperature Variation and Environment

It is worth mentioning that the experiments presented here that the DSC and X‐ray measurements were done in uncontrolled environmental conditions, PFM imaging was done in a controlled environment with dry‐air supply, and the *P*‐*E* spectroscopy was done for a sample with top and bottom electrodes. Moreover, one can expect that environmental effects are significant for lamellae, films, or particles^[^ ^34^, ^37^ ^]^ but less so for the bulk crystals that are presented in the current work. The highest temperature in the experiments was set to allow better control of the examined parameters: PE loop measurements were focused until 130 ⁰C, DSC, XRD‐RSM, and PFM measurements were set to a highest temperature of 160 °C, while dielectric measurements were performed for both 150 and 250 °C in independent measurements. To allow thermal shock in the TEM experiments, rapid cooling was performed from a maximum temperature of 800 °C. The asymmetric phase transition behavior was found to be highly reproducible over multiple heating and cooling cycles. Typically, the sample was held at ≈160 °C for ≈20 min after each heating, further confirming that the observed first‐order‐like transition during heating and second‐order‐like transition during cooling are intrinsic and unaffected by residual thermal strain effects. Note that phase‐transition signatures vary for the different physical properties. That is, crystal symmetry, heat flow, dielectric constant, and hysteretic behavior are expected to show a phase‐transition behavior at different temperatures.^[^ ^17^ ^]^

### DSC

Differential scanning calorimetry has been carried out with METTLER TOLEDO DSC 3+, STARe System, using a standard Aluminum metal reference with different heating and cooling rates (0.1–5 °C min^−1^).

### Impedance

The Dielectric measurement has been done with the MFIA Impedance Analyzer by Zurich Instruments with 0.3 V AC at 50 kHz connected to the MFP‐3D by Oxford Instruments’ Asylum PFM stage with precision temperature control (0.01 °C min^−1^). A capacitor geometry with the BaTiO_3_ crystal is used to measure the capacitance. The sample was glued on a thin mica substrate with silver paste (Ted Pella Inc) to ensure optimal thermal conductivity while preventing any electrical contact with the heater stage. Top and bottom circular gold electrodes (diameter 3 mm, thickness 80 nm) were fabricated with photolithography. The sample was mounted on the heater stage with the electrical connection to both the top gold and the bottom electrode. The heating and cooling rates were regulated through Oxford Instruments’ Asylum software. *T*_C_ of heating and cooling has been determined by the 90% fall of the maximum value of the dielectric permittivity.

### P‐E Loop

Temperature‐dependent *P‐E* spectroscopy was performed with the Precision Multiferroic Tester, Radiant Technologies, Inc. The hysteresis loop measurements were performed at 100 Hz with a double bipolar waveform at an amplitude of 20 V. Circular electrodes of 700 µm diameter of Ti (5 nm)/Pt (80 nm) metal were used to design the capacitor geometry. The sample on one side was affixed onto an aluminum nitride ceramic substrate (MTI Corporation) using silver paint (Ted Pella Inc.) to ensure optimal thermal conductivity while preventing any electrical contact with the Polyheater (a high‐temperature sample holder by Asylum Research). A custom‐made probing setup consisting of a microscope and micro‐positioner (Signatone Corp.) was employed to probe the devices for measurements. One probe tip was placed as a grounding point on the side of the sample, utilizing surplus silver paint that became available during the mounting process. The second probe tip was then positioned on the device under test for its electrical characterization throughout the heating and cooling cycles. The heating and cooling rates were regulated through software integrated with the MFP‐3D Origin AFM tool. The loops presented in Figure 2 represent the best possible measurements obtained near the Curie temperature (*T*_C_). At this temperature range, the polarization^[^ ^17^ ^]^ and coercive field^[^ ^39^ ^]^ both drop significantly. Moreover, near *T*_C_, the material exhibits increased leakage, which imposes the exertion of lower electric fields so that obtaining fully saturated loops becomes experimentally challenging. Nevertheless, the loops shown display clear flat regions at the extremes, a characteristic feature typically associated with saturation. These features, which indicate clearly on asymmetric behavior around the ferroelectric phase transition, thus provide meaningful insight into the ferroelectric response near *T*_C_, even in the absence of ideal saturation.

### PFM

Piezoresponse force microscopy is carried out in customized MFP‐3D by Oxford Instruments’ Asylum with precision temperature control (0.01 °C min^−1^) at ambient conditions. ADAMA tip AD‐2.8‐AS with a spring constant of 3.73 has been used. The images were taken in vector mode at a vertical frequency of 349.76 kHz and a lateral frequency of 749.92 kHz. Drive amplitude for both vertical and lateral excitations was 1 V. All PFM and AFM images were taken at 256 × 512 pixel‐resolution scans, using Asylum Research's software. For all images, both the backward and forward scans were recorded, while here, only the forward‐direction images are presented for the sake of consistency. No manipulation has been conducted on the AFM and PFM images other than the usual AFM flattening that compensates for the tip scan.

### RSM

Reciprocal Space Mapping is done in the BaTiO_3_ crystal at the custom‐built four‐circle X‐ray diffractometer, equipped with a microfocus X‐ray source, double‐crystal monochromator, Eulerian cradle, a PILATUS 1 m pixel area detector, and Forvis‐technologies fine temperature control sample environment cell. The experiment began with the determination of the crystal orientation with respect to the diffractometer axes. Then the crystal was rotated to the specific orientation, which realizes the Bragg condition for the specific reflection. Then, the diffraction intensity distribution around each reflection was measured with high angular resolution and represented as a function of the reciprocal space coordinates. This way, reciprocal space volumes (RSV) were collected around selected Bragg peaks with very fine (0.05 °C) temperature steps, both on heating and cooling. A series of 3D RSVs were collected, which represent diffraction intensity distribution around certain families of Bragg peaks 002, 102, 104, and 222. Such Bragg peaks split into several components due to the presence of ferroelastic domains.

### Phase‐Field Modeling

The phase‐field simulations were performed to evaluate the energy and polarization changes for a bulk BaTiO_3_ single crystal under a macroscopically stress‐free state during one slow cooling and heating cycle. To mimic the quasi‐static variation of temperature, the kinetics of the ferroelectric system is described by the time‐dependent Ginzburg–Landau (TDGL) equation, $\frac{\partial P(x,t)}{\partial t}=-L\frac{\delta F}{\delta P(x,t)}+\xi \left(t\right)$, where ***P*** (***x***, *t*) is the total polarization field at time *t*, *F* the total free energy of the system, *L* the kinetic coefficient which is assumed to be isotropic, and the Langevin noise term ***ξ***. The total free energy *F* contains contributions from the bulk free energy modeled by an eighth‐order Landau‐type polynomial, the elastic strain energy accounts for the strain‐polarization coupling, the electrostatic energy for the dipole–dipole interactions, and the gradient energy associated with the inhomogeneities of polarization across the domain walls and ferroelectric‐paraelectric phase boundaries. Detailed expressions of these energy terms have been well documented and can be found in ref. [40]. The Langevin noise ***ξ*** is considered to enable the nucleation of ferroelectric phases within the cubic parametric phase upon cooling. To implement it, random noise polarization Δ ***P*** (***x*** *, t*) was applied at each time step with a uniform distribution of the magnitude |Δ ***P*** | between zero to |Δ ***P*** |_max_, and for simplicity, |Δ ***P*** |_max_ = 0.01 C m^−2^ was assumed and is temperature independent. During the cooling and heating simulations, the temperature is assumed to be uniform across the system and varies linearly from 428 to 298 K during cooling and rises from 298 K back to 428 K during heating. The total timesteps for the cooling and heating are 100 000 respectively, to allow sufficient relaxation of the system to the quasi‐equilibrium. The BaTiO_3_ bulk crystal is modeled as a quasi‐2D system with 128 nm‐by‐128 nm in the *x*‐*y* directions and the *z* direction periodic. The *x*, *y*, and *z* directions in the simulation correspond to the [100] and [011], and [01$\bar{1}$] axes of the cubic BaTiO_3_. The reason for the rotation of the coordinate system is to ensure the nucleation of 90° ferroelastic domain walls by eliminating the constraint imposed by the periodic boundary condition in the *z* direction. Otherwise, only 180° ferroelectric domain walls will form if the *x*, *y*, and *z* directions are parallel to the [100] and [010], and [001] directions. The stereographic projection of the local polarization vectors onto the pole figure follows the method used in ref. [41], and all the polarization vectors are normalized by the stress‐free spontaneous polarization | ***P***_0_| = 0.26 C m^−2^ at *T* = 298K. The Landau coefficients used for the thermodynamic analysis of BaTiO_3_ in Figure 5A are adopted from the 6th order.^[^ ^27^ ^]^ for deriving a closed‐form analytical expression of ***P*** and the equilibrium free energy as a function of the temperature, while the Landau coefficients used for the phase‐field simulations are adopted from the 8th‐order model for numerical stability considerations. The comparison in Figure 5 is achieved by normalizing the free energy calculated from phase‐field simulations to the analytical theory (i.e., shifting the temperature by 5 K and the energy density by a constant factor). Note that this normalization is made for better numerical comparison and does not affect the conclusions. The phase‐field simulation is performed using the ferroelectrics module of the commercialized software µ‐Pro.

## Conflict of Interest

The authors declare no conflict of interest.

## Author Contributions

A.H. and E.H. contributed equally to this work. A.H. performed PFM measurements, analyzed and organized the data, managed the whole project, and helped write the manuscript. E.H. managed the project, analyzed the data, discussed, and helped write the manuscript. R.M. has performed DSC measurements and helped write the manuscript. P.K. has done the PE loop tracing with the help of CCL. J.T. has performed dielectric measurements. M.B. has done the STEM experiment. S.G. has analyzed the crystallography data and performed the RSM measurements with the help of D.S. and A.H. I.V.J. and N.D. have helped in the discussion and writing the manuscript. L.Q.C. helped analyze the data as well as perform the phase‐field simulations with B.W. L.W.M. has supervised the P‐E measurements, helped analyze the data, and hosted Y.I. during this work. Y.I. initiated the research, helped analyze the data, and wrote the manuscript. A.H. and H.E. have contributed equally to this work. All authors have contributed to the manuscript preparation and read the final manuscript.

## Supporting information



Supporting Information

Supplemental Video 1

Supplemental Video 2

Supplemental Video 3

Supplemental Video 4

Supplemental Video 5

Supplemental Video 6

Supplemental Video 7

Supplemental Video 8

## Data Availability

The data that support the findings of this study are available from the corresponding author upon reasonable request.
