# Supplementary material for: Asymmetry of the Ferroelectric Phase Transition in BaTiO3
Source: Adv Mater. 2025 Dec 10;38(25):e16507. doi: 10.1002/adma.202516507 (PMC13137769; doi:10.1002/adma.202516507)
Supplement: Supplementary file 1 — Supporting Information [file ADMA-38-e16507-s005.pdf]

Supplementary Information for "*Asymmetry of the Ferroelectric Phase Transition in $BaTiO_3$*"

Asaf Hershkovitz,[1,2*] Elangovan Hemaprabha,[3]* Rajesh Mandal,[1,2] Pravin Kavle,[4,5] Jamil Tanus,[2] Maya Barzilay[1,2] Ching-Che Lin,[4] David Spirito,[6] Semën Gorfman,[6] Bo Wang[7,8], Ignacio J. Villar-García,[10] Neus Domingo[9,11], Long-Qing Chen[8], Lane W. Martin,[4,5,12] and Yachin Ivry[1,2,4,13†]

1. Technion Israel Institute of Technology, Department of Materials Science and Engineering, Haifa 3200003, Israel
2. Technion Israel Institute of Technology, Solid State Institute, Haifa 3200003, Israel
3. Department of Metallurgical and Materials Engineering, Indian Institute of Technology Madras, Chennai 600036, India
4. Department of Materials Science and Engineering, University of California, Berkeley, CA 94720, USA
5. Materials Sciences Division, Lawrence Berkeley National Laboratory, Berkeley, CA 94720, USA
6. Department of Materials Science and Engineering, Tel Aviv University, Wolfson Building for Mechanical Engineering, Tel Aviv, 6997801, Israel
7. Materials Science Division, Lawrence Livermore National Laboratory, California 94550, USA
8. Department of Materials Science and Engineering, The Pennsylvania State University, University Park, PA 16802, USA
9. ICN2 - Institut Catala de Nanociencia i Nanotecnologia, Campus UAB, 08193 Bellaterra (Barcelona), Spain
10. CELLS-ALBA Synchrotron Radiation Facility, Cerdanyola del Valles, Spain
11. Center for Nanophase Materials Sciences, Oak Ridge National Laboratory, Oak Ridge, TN 37830, USA
12. Departments of Materials Science and Nano Engineering, Chemistry, and Physics and Astronomy and the Rice Advanced Materials Institute, Rice University, Houston, TX 77005, USA
13. The Nancy & Stephen Grand Technion Energy Program (GTEP), Technion − Israel Institute of Technology, Haifa 3200002, Israel

* These authors contributed equally to this work

† Correspondence to: ivry@technion.ac.il

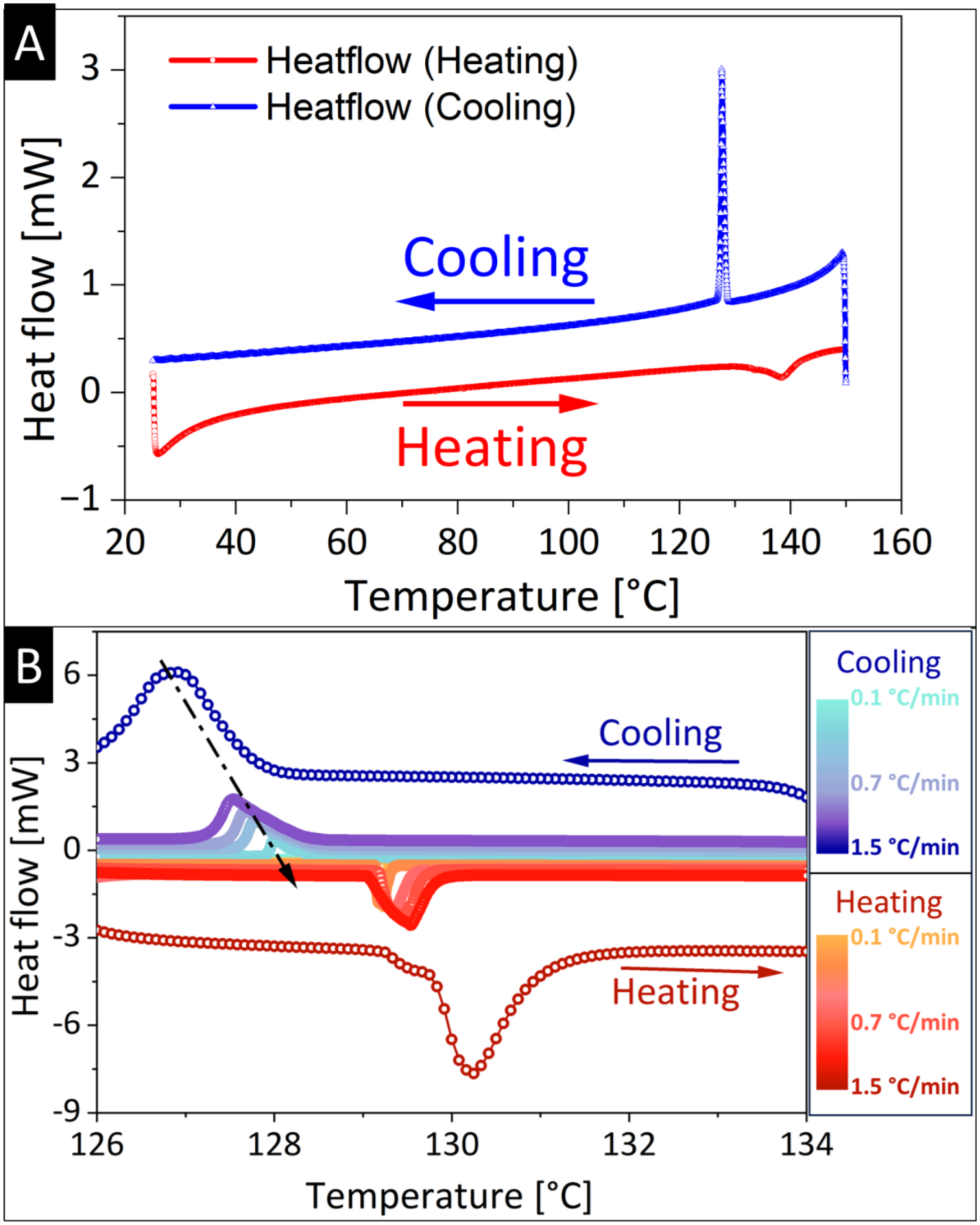

Figure SI1| Differential scanning calorimetry (DSC) heat flow measurements at various scan rates between, during both heating and cooling for $BaTiO_3$. (**A**) DSC data for heating and cooling cycle from broad temperature range. (**B**) Difference in the transition temperatures (ΔT_{on-off}) during heating and cooling for different temperature variation rates (0.1 °C/min - 1.5 °C/min) are extracted from these measurements and provided in Figure 1D.

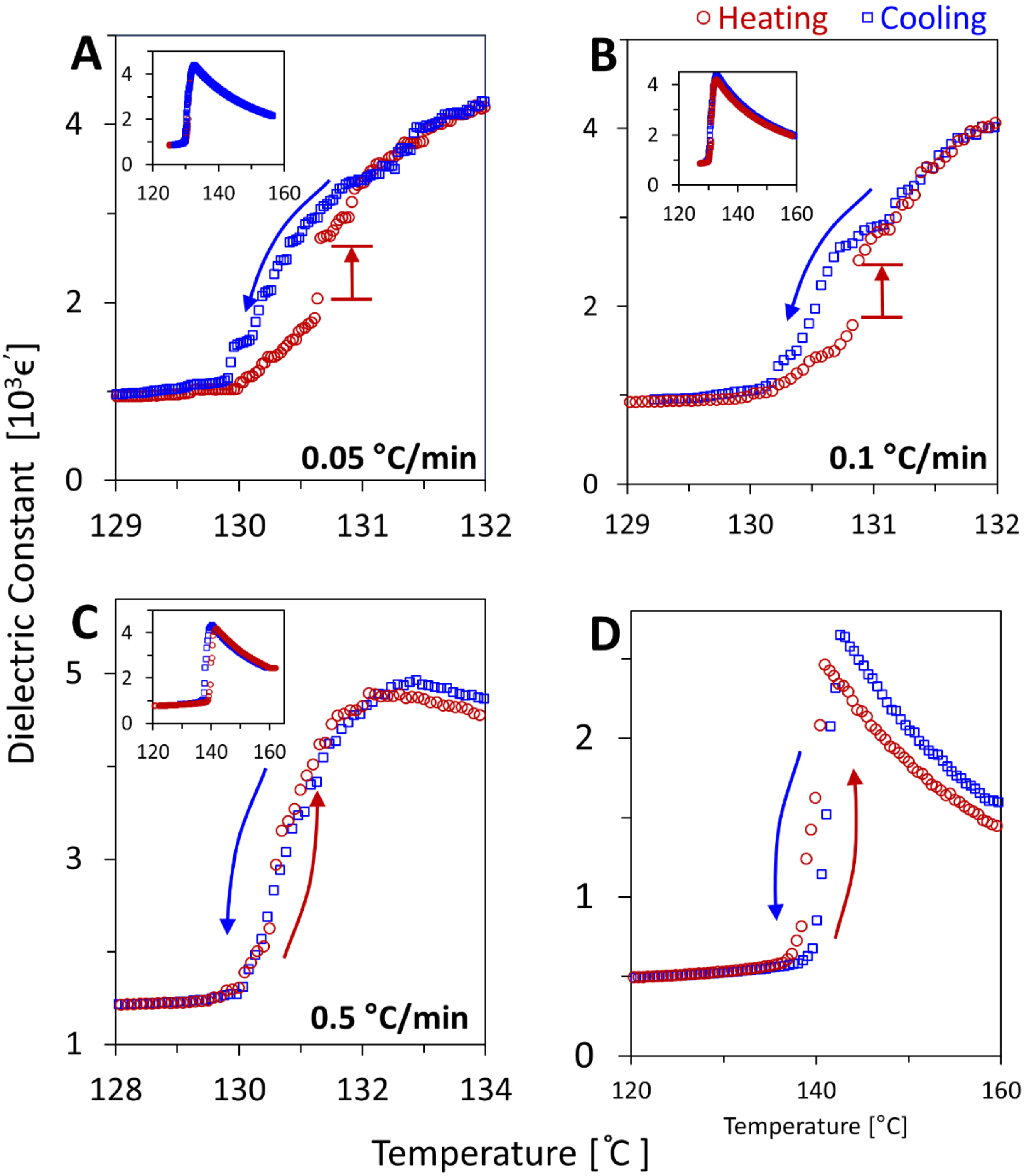

Figure SI2| Dielectric permittivity as a function of temperature at various scan rates. Dielectric permittivity at heating (red) and cooling (blue) at temperature variation rates of (A) 0.05, (B) 0.1, (D) 0.5 and (E) 2 °C/min. During slower temperature variation rates (A-B), the dielectric permittivity decreased smoothly upon cooling, indicating on a second order-like transitions (note that a shoulder at the transition may be an indication of an intermediate state that mediates the transition). Contrariwise, first-order transition was observed upon heating, where the dielectric permittivity exhibits a sudden jump (highlighted). For faster temperature variation rates (C-D), the transition was symmetric and gradual, indicating on a second order-like transition. Inserts include the full range scan of the dielectric permittivity for each temperature variation rate.

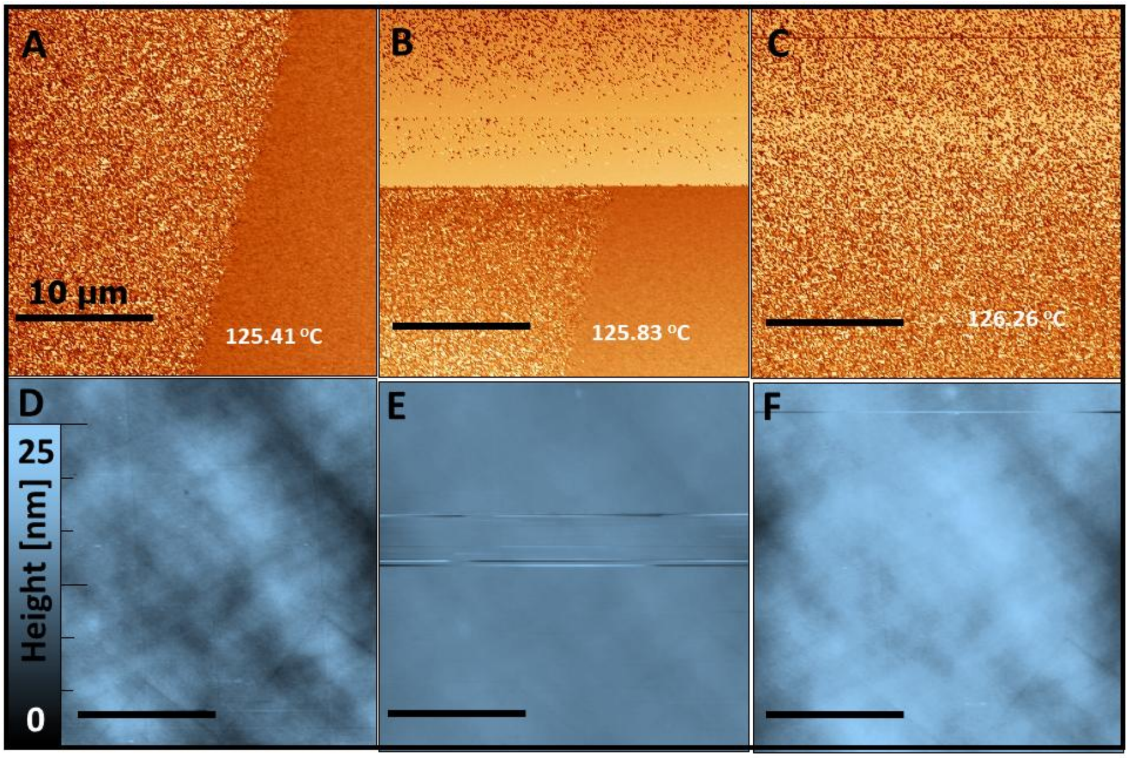

Figure SI3| PFM vertical amplitude (**A**, **B** & **C**) and topography (**D**, **E** & **F**) during heating.

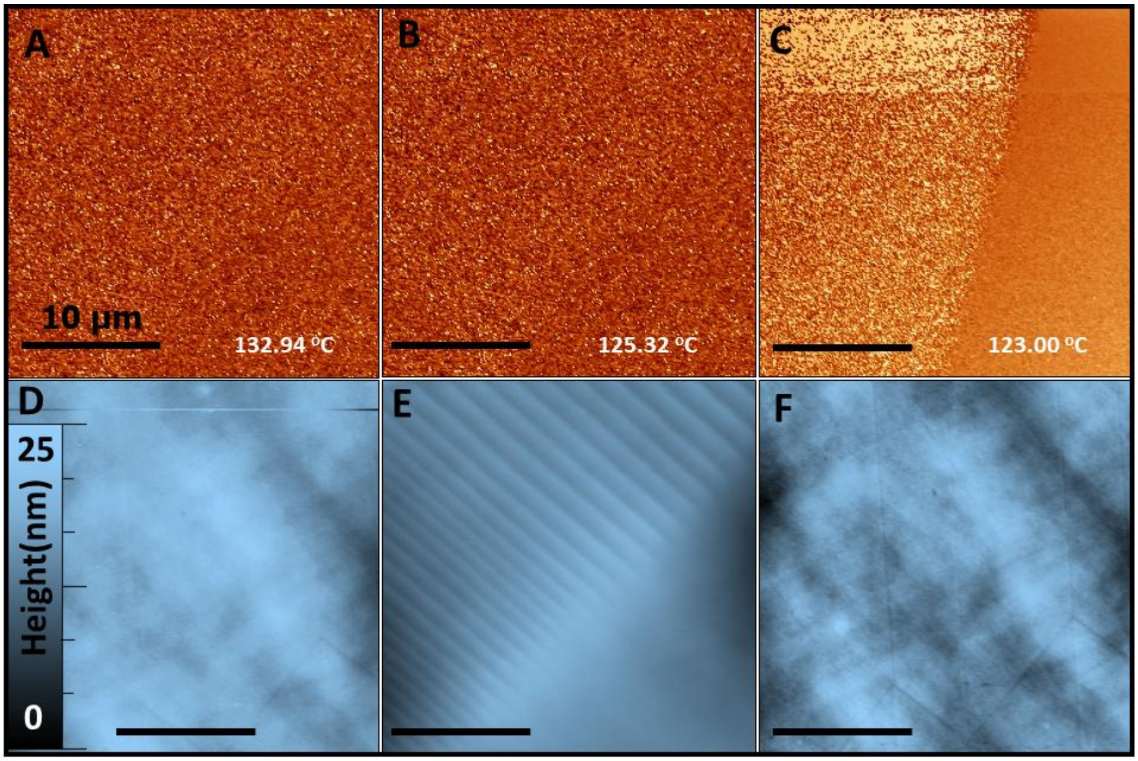

Figure SI4| PFM vertical amplitude (**A**, **B** & **C**) and topography (**D**, **E** & **F**) during cooling.

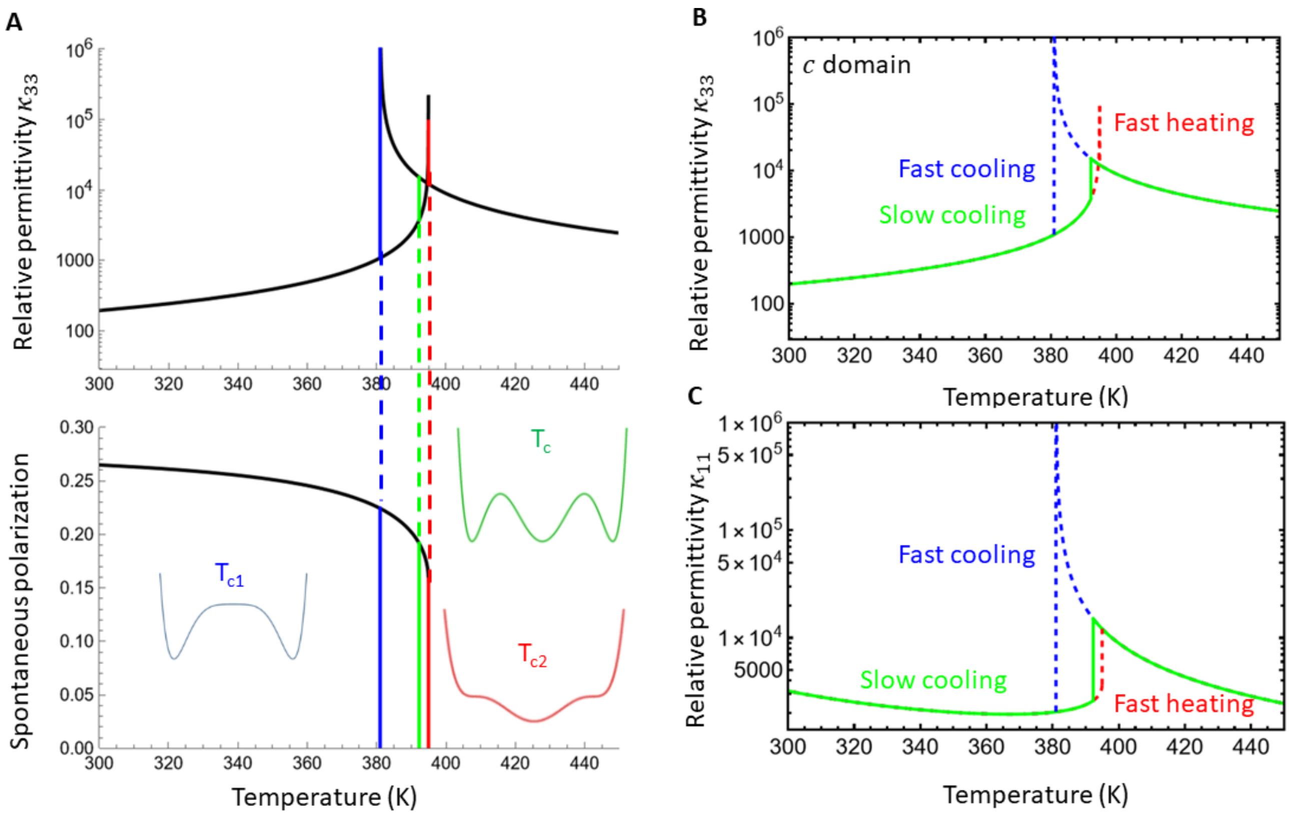

Figure SI5| Thermodynamic calculation of the spontaneous polarization and relative permittivity at equilibrium as a function of temperature for single-domain BTO. (**A**) The spontaneous polarization and relative permittivity at equilibrium as a function of temperature. The blue line corresponds to the lowest temperature (T_{C1}) for the paraelectric cubic phase being metastable above the Curie temperature T_C while the red line corresponds to the maximal temperature (T_{C2}) for the ferroelectric tetragonal phase being metastable above T_C. The insets in the lower panel indicate the free energy landscape for the different temperatures. (**B**) A replot of upper panel of (**A**) demonstrating the behavior of the longitudinal relative dielectric permittivity κ_{33} under virtually fast/slow heating and cooling. (**C**) The same plot as (**B**) but for the transverse relative dielectric permittivity κ_{11}.

SI5: Once the asymmetric behavior at the phase transition was established experimentally both at the macroscopic and microscopic scales, a deeper discussion about the origin of this effect was needed. To understand the asymmetric behavior of the phase transitions and the possible mechanism for the formation of intermediate phase upon cooling, a sixth-order Landau-Ginzburg-Devonshire (LGD) theory[1] for a homogeneous monodomain ferroelectric was used. The free energy change of a uniaxial ferroelectric with respect to the paraelectric phase under zero electric field is thus given by:

$$\Delta f = f_{\mathrm{FE}} - f_{\mathrm{PE}} = \frac{1}{2}\alpha P^2 + \frac{1}{4}\beta P^4 + \frac{1}{6}\gamma P^6, \quad (1)$$

where α, β, and γ are coefficients which vary with temperature, stress and strains. P serves as the order parameter, and its value at equilibrium state can be determined by minimizing Δf with respect to P.[2] According to the LGD theory, a ferroelectric transition is first order when $\beta < 0$ and $\gamma > 0$, while it is second order when $\beta > 0$.[2] It is also known that strain can modify the sign of β and thus alter the phase transition order.[3] Using the sixth-order model with coefficients from Ref.[4], for stress-free single-domain $BaTiO_3$, we calculated the equilibrium P and the dielectric permittivity κ_{11} and κ_{33} as a function of temperature (Figure SI5). We find a strong asymmetry in terms of the superheating ($\Delta T_h = T_{C2} - T_C$) and undercooling ($\Delta T_C = T_C - T_{C1}$, the definitions of T_C, T_{C1} and T_{C2} are described in SI), *i.e.*, $\Delta T_C >> \Delta T_h$. This difference suggests that the paraelectric-ferroelectric phase coexistence can occur at a wider temperature range upon cooling than upon heating, in agreement with the slow cooling-rate DSC results (Figure 1A). The temperature dependences of both κ_{11} and κ_{33} show λ-shaped behavior with a finite discontinuity at the transition temperature, a key characteristic for first-order transitions.[2] Notably, when the phase transition occurs at the quasi-static limit, the discontinuity of the dielectric permittivity is much smaller than that in the undercooling or superheating regimes. This finding explains the difference in the measured dielectric permittivity between Figures 1E and F.

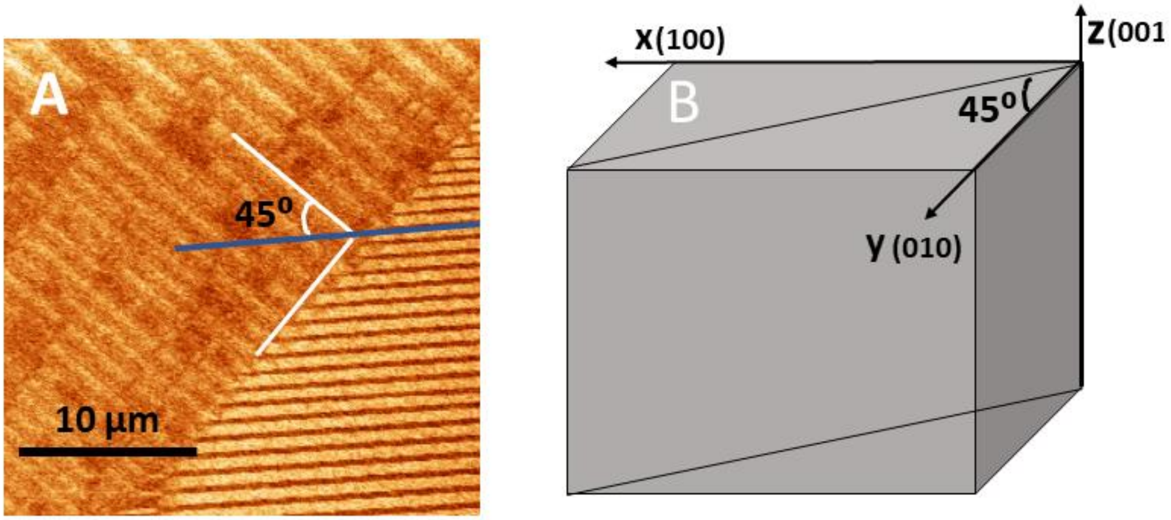

Figure SI6| Geometry of the intermediate domain phase showing an angle of 45° with the striped ferro elastic domains of the final tetragonal phase (**A** & **B**).

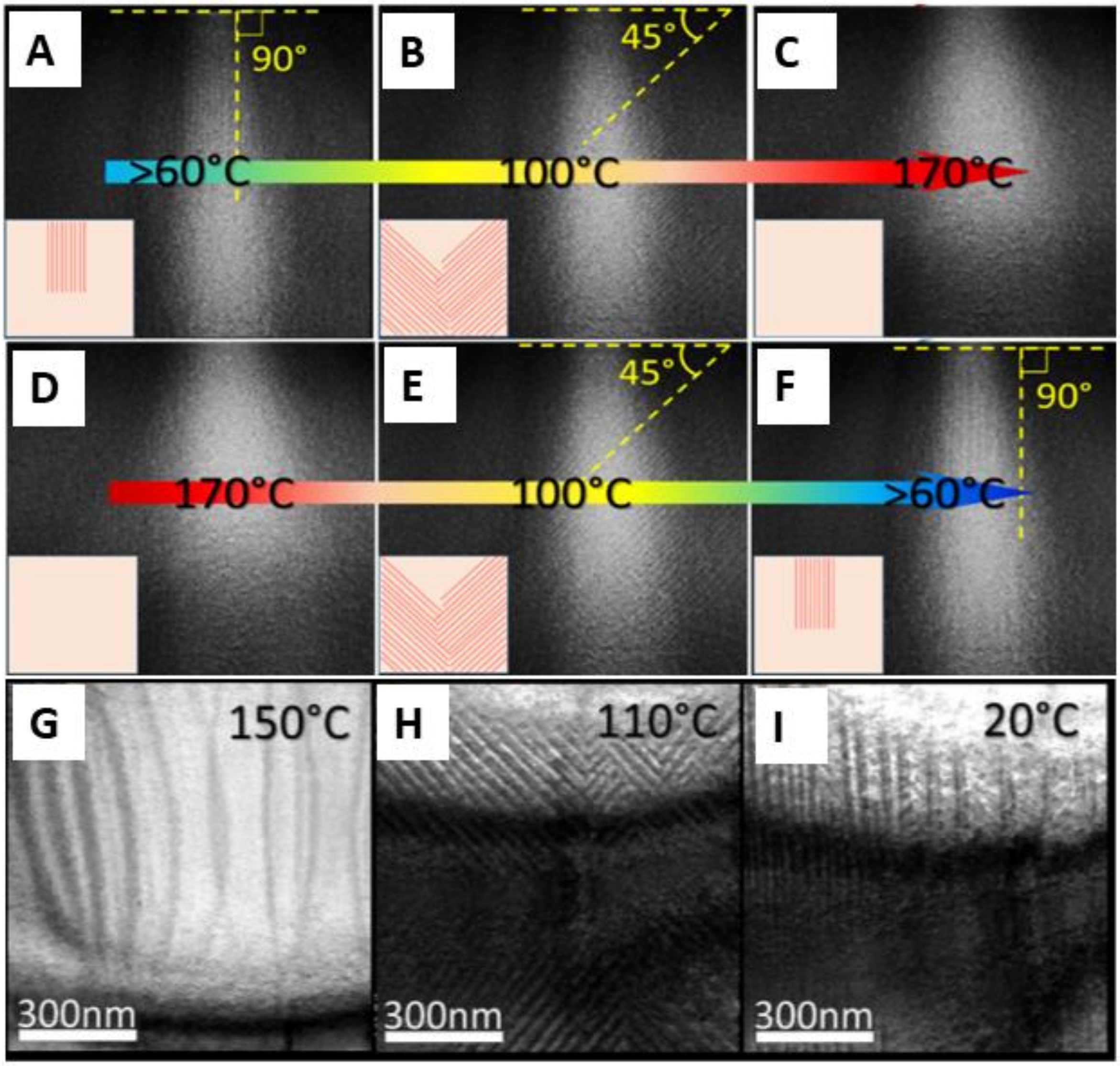

Figure SI7| Heating & Cooling experiment with $BaTiO_3$ lamella in STEM-HAADF. (**A-F**) Domain rotation at elevating temperatures. (**A**) Below 60 °C, only vertical domains, perpendicular to the surface of the single crystal, appear. (**B**) Above 60 °C, diagonal domains appeared and the vertical domains disappeared. (**C**) Above T_C, all domains were eliminated as the material crystallographic structure became cubic. During cooling, the domains were stable at the same temperature range, whereas (**D**) above T_C, no domains were detected. (**E**) Between T_C and 60°C, only diagonal domains that were oriented to 45° to the single crystal surface existed. (**F**) Below 60 °C, the vertical domains reappeared. (**G-I**) Domain rotation with rapid cooling after thermal shock. Before thermal shock and above T_C, at 150 °C, no domains appeared, only band contours (**G**). After the rapid cooling from 800 °C, a bundle of diagonal domains appeared at 110°C (**H**). Only below 30°C the vertical domains reappeared (**I**).

SI7: $BaTiO_3$ crystals are under stress-free condition macroscopically in the experiments. Yet, strain can be inhomogeneous locally, *e.g.*, surfaces can be stress free, while the interiors are constrained. The calculations show (Figure 5A) that the cubic-to-tetragonal phase transition of $BaTiO_3$ is first-order under stress-free condition. However, it becomes second-order when tri-axial strain conditions are introduced. Similar to a martensitic transition, the strain itself is asymmetric in $BaTiO_3$ for transitioning from and to the ferroelectric (tetragonal) state. Therefore, a mixed phase transition behavior is expected. The strain state can also alter the relative stability of different ferroelectric phases. For example, an orthorhombic monodomain under the tri-axial strains is more stable than the a tetragonal monodomain (Figure 5A). This observation supports the existence of the observations of an intermediate phase at certain temperature and local strain conditions.

The presented thermodynamic analyses introduce possible mixed behavior of first- and second-order like phase transitions along with the presence of lower-symmetry intermediate phases, both of which are mediated by the elastic strain energy. To further verify these theoretical insights, phase-field simulations were performed for phase transitions upon heating and cooling of inhomogeneous $BaTiO_3$ bulk crystals that are subject to macroscopically stress-free conditions without the assumption of monodomain state. A quasi-2D model of $BaTiO_3$ (128 nm × 128 nm × 1 nm) with three-dimensional periodic boundary conditions was established (see Methods for details). The time-dependent Ginzburg-Landau equation was solved to obtain the equilibrium polarization at various temperatures. Starting from a paraelectric phase at an initial temperature T_{init} = 428 K, the temperature was decreased to T_{fin} = 298 K at a slow rate to mimic the quasi-static cooling and then raised the temperature back to T_{init} at the same rate to mimic the quasi-static heating. To break the homogeneity and facilitate the nucleation of a ferroelectric phase during cooling, we introduce a random noise distribution of polarization with small magnitudes. During the cooling and heating simulations, the total free energy and the elastic energy densities were calculated as a function of temperature (upper and lower panels of Figure 5A, respectively). A strong asymmetry between the transition to and from the ferroelectric state was found. The transition upon heating shows an abrupt change at T = 395 K and a narrow overheating regime (~2 K as indicated by dashed red lines) while the transition upon cooling tends to be diffuse with a ~18 K undercooling regime as indicated by dashed blue lines. This behavior is in good agreement with the DSC measurements in Figure 1. The total-free-energy variation during the heating simulation matches very well with the analytical monodomain results and the elastic strain energy remains small and invariant, suggesting a homogeneous transition of the system during heating without nucleation and growth of the cubic phase. In contrast, the change of total free energy during cooling does not follow any of the monodomain energies and go across the

metastable region of the orthorhombic phases, suggesting an inhomogeneous nucleation and growth process involving intermediate phases. Meanwhile, the corresponding elastic strain energy shows a peak, suggesting that, unlike the heating process, the nucleation of a ferroelectric phase within the paraelectric matrix requires to overcome a strain energy barrier. This difference is essential for understanding the asymmetry behavior.

To reveal the intermediate-phase structure, domain structure was simulated at $T = 377$ K during cooling (Figure 5D) and the statistical distribution of P was visualized by stereographic projection (Figure 5E). The corresponding equilibrium domain structure and pole figure at $T = 298$ K are given in Figure 5B-C as a reference. The volume fractions of tetragonal, intermediate, and cubic phases during the cooling and heating simulations are shown in Figure 5F. It was found that upon cooling from the cubic state, intermediate phases form at high amount (up to ~ 20% in volume fraction) at junctions of the tetragonal domain variants and disappear as the cooling proceeds. This observation suggests that the intermediate phases form to accommodate the polarization and strain mismatch between the nucleated tetragonal domain variants. The slight increase of the intermediate phase fraction upon heating is due to the broadening of the domain walls which has a lower symmetry than the tetragonal domains. Therefore, the phase-field simulations not only comply with the asymmetric behavior of the phase transitions seen in the experiments but also evidence the formation of intermediate phases as a mechanism to accommodate the nucleation of ferroelectric tetragonal domain variants within the paraelectric cubic matrix. While the exact role of the intermediating phase during slow cool-down is currently not completely understood, the combined experimental – phase-field data suffice to propose a preliminary explanation.

SI8: The lamella was prepared by high energy ions in a FIB (FEI Helios NanoLab DualBeam G3 UC, Ga+), and they were attached to a DENSsolutions Nano-Chip. Because the lamella was expected to slightly bend, it was imaged by STEM- HAADF (aberration-corrected Titan Themis 80–300 operated at 200 kV), which is less sensitive to bent contours. Heating & cooling experiment has been done a step of 5 °C every 2 min (Figure SI7 A-F). The influence of a thermal shock on the lamellae has been studied by a fast heating and cooling procedure, creating a temperature peak from 135°C to 800°C and back within less than 2 minutes (Figure SI7 G-I).

As discussed in the paper, this observation also could be well understood with the phase diagram in the thermal-elastic energy space developed by Koukhar et al.[10] Because the examined lamellae did not go through an annealing process, it most likely contains residual strain, which in turn can be the reason for the observed domains rotation at elevated temperatures. Evaluating the strain in our samples according to the misfit strain-temperature phase diagrams suggests strain of $0.9 \cdot 10^{-3}$ (dashed orange lines in Figure SI8). High thermal shock also responsible for inducing a high amount residual strain in the system that sustains in a long temperature range until room temperature (<30°C) as observed from the stable bundle of diagonal domains (Figure SI7 H).

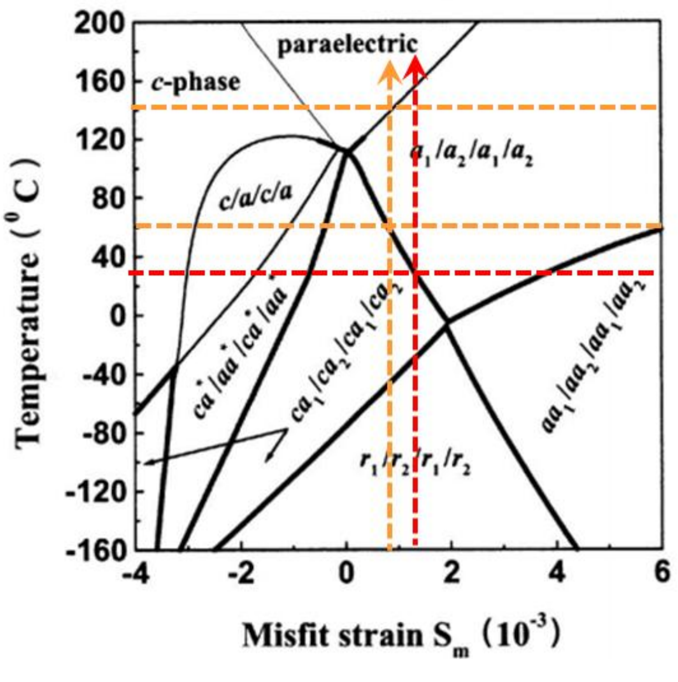

Figure SI8| Calculated ''misfit strain-temperature'' phase diagrams of $BaTiO_3$ epitaxial films, grown on cubic substrates. The horizontal orange dashed lines mark the transformation temperature observed in our $BaTiO_3$ lamella. The orange arrow marks the equivalent strain in our samples. The horizontal red dashed lines mark the transformation temperature after thermal shock. The red arrow marks the equivalent strain. Image taken from Ref.[10]

Videos SI1-8| Temperature-dependent XRD-RSM data. Videos SI1-8 show the temperature evolution of the 3D reconstruction of the RSM data obtained from different symmetric and asymmetric crystallographic orientations (labeled in the Videos), allowing comprehensive analysis of the phase dynamics around the ferroelectric phase transition. Specifically, Videos SI1-8 show the RSM data of alternating cooling and heating experiments obtained from the (002), (102), (104) and (222) orientations, respectively.

Note that the reason it may appear that peak a2 (or other peaks) looks asymmetric is that the actual X-ray scattering intensity is represented as a function of three coordinates (B_x, B_y, B_z). Figure 4H shows the projection of the intensity distribution along the Y-axis, while Figure 4O shows a similar projection along the Z-axis. These projections result in the apparent overlap of peaks with different coordinates along the projection axis. What appears as a shoulder of the a2 peak in Figure 4H is actually the a1 peak. Another projection of the same intensity distribution (Figure 4O) demonstrates that the peaks are well-separated, with none displaying any significant asymmetry.

The intensity of a particular sub-peak depends on the volume of the corresponding domains (in this case, a1) within the X-ray beam. It is important to examine the specific XZ projection in Figure 4C in conjunction with the XY projection in Figure 4J for the same peak. As shown in Figure 4J, there are two peaks associated with a1 domains. These two peaks share the same B_x and B_z coordinates, causing them to appear overlapped in the XZ projection. This overlap contributes to the seemingly high intensity of the peak.
